# Loss of sheen in Oguchi disease following short wavelength exposure

**DOI:** 10.1038/s41433-024-03237-4

**Published:** 2024-07-14

**Authors:** Haseeb N. Akhtar, Luke Nicholson, Zoe Ockrim, Magella Neveu, Andrew R. Webster, Michel Michaelides, Omar A. Mahroo

**Affiliations:** 1 UCL Institute of Ophthalmology, University College London, London, UK; 2 Retinal Service, Moorfields Eye Hospital, London, UK; 3 Electrophysiology Service, Moorfields Eye Hospital, London, UK; 4 Department of Ophthalmology, St Thomas’ Hospital, London, UK; 5 Section of Ophthalmology, King’s College London St Thomas’ Hospital Campus, London, UK; 6 Translational Ophthalmology, Wills Eye Hospital, Philadelphia, USA; 7 Physiology, Development and Neuroscience, University of Cambridge, Cambridge, UK

**Keywords:** Retina, Retinal diseases

In Oguchi disease [1], patients experience lifelong nyctalopia, and a striking golden retinal sheen is apparent on fundus examination, which disappears following prolonged dark adaptation (Mizuo-Nakamura phenomenon) [2]. The condition arises from biallelic variants in the *GRK1* or *SAG* genes, which encode rhodopsin kinase and arrestin respectively [3, 4]. Here, we report a phenomenon where, paradoxically, loss of the sheen occurs following bright short wavelength illumination (i.e. following illumination rather than following prolonged dark adaptation). We examined colour (Clarus, Zeiss) or pseudocolour (Optos plc) fundus images from patients with a clinical and/or genetic diagnosis of Oguchi disease, noting whether loss of the sheen was observable centrally, corresponding to the area illuminated by prior 30-degree 488 nm autofluorescence (AF) imaging (Spectralis, Heidelberg). Figure 1 shows this clearly in two patients: a square-shaped loss of sheen is evident. Both patients had undergone prior 30-degree 488 nm AF imaging.

**Fig. 1:**
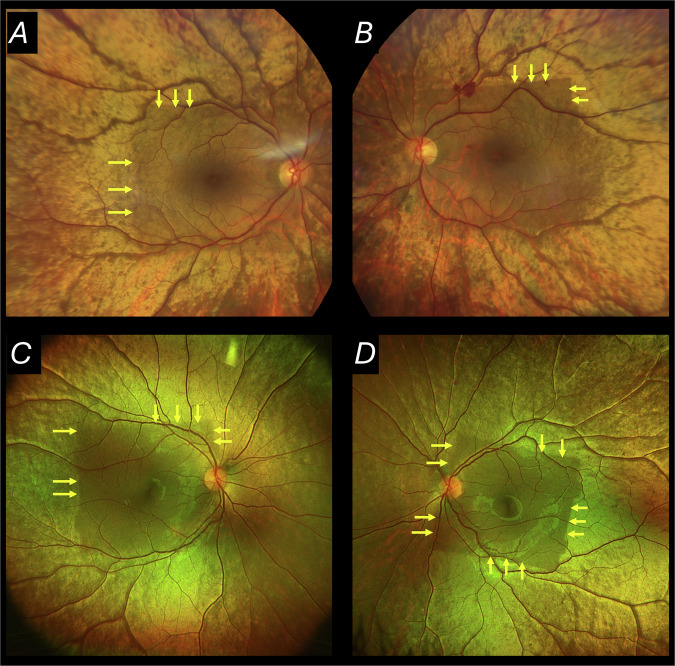
Absence of sheen in area of prior 488 nm AF imaging. Fundus images from 2 patients with Oguchi disease in whom 30-degree short wavelength AF images had been taken some minutes previously. **A**, **B** Colour images (Clarus, Zeiss) in a 67-year-old patient with a clinical diagnosis of Oguchi disease (this patient also had a small retinal haemorrhage in the left fundus of unknown cause). **C**, **D** Pseudocolour images (Optos) from a 12-year-old patient with molecularly proven Oguchi disease (biallelic variants in *GRK1*). A central square of reduced sheen is evident in both eyes of both patients (borders of the squares are partly highlighted by yellow arrows).

To test the hypothesis that this was attributable to the prior AF imaging, we performed AF imaging in a third patient initially only in one eye, followed by pseudocolour imaging in both eyes. This is shown in Fig. 2, where the central loss of sheen is only evident monocularly. When pseudocolour imaging was then repeated following bilateral AF imaging, the loss of sheen was evident in both eyes. The phenomenon was also observable on clinical fundus examination.

**Fig. 2:**
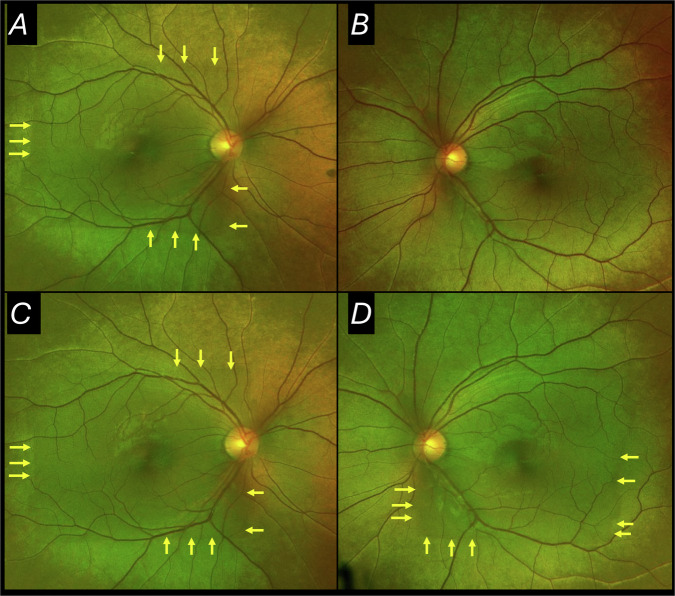
Absence of sheen depends on prior 488 nm AF imaging. **A**, **B** Optos pseudocolour images taken from both eyes of a 28-year-old patient with molecularly proven Oguchi disease (biallelic variants in *GRK1*) following prior AF imaging in the right eye only. The central square of reduced sheen is evident only in the right eye. **C**, **D** Optos pseudocolour images taken from both eyes of the same patient following prior AF imaging both eyes. The central square of reduced sheen is evident now in both eyes. In (**A**, **C**, **D**), borders of the square are partly highlighted by yellow arrows.

In Oguchi disease, the shut-off of light-activated rhodopsin is impaired. It is possible that the presence of substantial light-activated rhodopsin (unphosphorylated or uncapped by rhodopsin kinase or arrestin respectively) directly gives rise to the well-described sheen. The bright blue 488 nm light used in short wavelength AF imaging will bleach a significant proportion of rhodopsin, which will take several minutes to regenerate (owing to the time taken for retinoid recycling). Thus, our present findings suggest that the presence of large amounts of regenerated rhodopsin is also a precondition for the Oguchi sheen. This hypothesis is supported by our prior observation of loss of the sheen in an area of neurosensory retinal detachment in a patient with Oguchi disease and central serous retinopathy [5]. Here, also retinoid recycling (which occurs via the retinal pigment epithelium) is expected to be disturbed. These findings yield new insights into aspects of the sheen in Oguchi disease; they might also have relevance to other conditions characterized by retinal sheens.

## Data Availability

The anonymised data processed for the analysis in this manuscript are available on request.
